# Molecular Dynamics Simulations of Mite Aquaporin DerfAQP1 from the Dust Mite *Dermatophagoides farinae* (Acariformes: Pyroglyphidae)

**DOI:** 10.1155/2020/6717390

**Published:** 2020-07-23

**Authors:** Li-lei Wang, Li-li Yu, Ying Zhou, Mei-li Wu, Fei-xiang Teng, Nan Wang, Yu-bao Cui

**Affiliations:** ^1^Shandong Institute of Parasitic Diseases, Shandong First Medical University & Shandong Academy of Medical Sciences, Jining, 272033 Shandong Province, China; ^2^Department of Medical Technology, Jiangsu Vocational College of Medicine, Yancheng, 224006 Jiangsu Province, China; ^3^Department of Pediatrics Laboratory, Wuxi Children's Hospital, Wuxi, 214023 Jiangsu Province, China; ^4^Department of Clinical Laboratory, Wuxi People's Hospital Affiliated to Nanjing Medical University, Wuxi, 214023 Jiangsu Province, China

## Abstract

Aquaporins are a large family of transmembrane channel proteins that facilitate the passive but highly selective transport of water and other small solutes across biological membranes. House dust mite (*Dermatophagoides farinae*) is the major source of household immunogens, and we have recently reported six cDNA sequence encoding aquaporins from this mite species. To better understand the structure and role of mite aquaporin, we constructed a tertiary structure for DerfAQP1 by homology modeling from the X-ray structure of malaria aquaporin PfAQP (Protein Data Bank code No. 3C02) and conducted molecular dynamics simulation. The simulation arranged seven water molecules in a single file through the pores of the DerfAQP1. Further, two conserved Asn-Pro-Ala motifs were located on Asn203 and Asn77; residues Arg206, Trp57, Met190, Gly200, and Asp207 constituted an extracellular vestibule of the pore; and residues His75, Val80, Ile65, and Ile182 constituted the cytoplasmic portions. The overall free energy profile for water transport through DerfAQP1 revealed an energy barrier of ~2.5 kcal/mol. These results contribute to the understanding of mite physiology and pathology.

## 1. Introduction

House dust mites are a worldwide health problem and are a major risk factor for asthma, rhinitis, dermatitis, and other allergic disorders. More than 15%–20% of the populations in industrialized countries are affected by house dust mite sensitization [1]. The two most important elicitors of mite allergies are house dust mites *Dermatophagoides pteronyssinus* and *D. farinae*, which are both members of the Pyroglyphidae family. The prevalence of these two major species of mites depends on the geographical region, with *D. pteronyssinus* as the most prevalent species in Europe, North and South America, Africa, and Australia. Meanwhile, *D. farinae* is often misleadingly described as the American house dust mite, although it is dominant only in the northeastern parts of North America [2]. Chemicals have been tested in the laboratory for their ability as acaricides to kill house dust mites, but most have poor control or 100% mortality at very high concentrations, thus posing a threat to the environment and humans.

Aquaporins (AQPs) are a family of small, integral membrane proteins that play critical roles in facilitating water transport across cellular plasma membranes in response to osmotic gradients. AQP performs fundamental tasks in the physiology of virtually every living organism, including higher mammals, other vertebrates, invertebrates, plants, eubacteria, archaebacteria, and other microbes. Proteins are involved in diverse biological processes throughout the natural world, making them attractive targets for the development of novel drug therapies or inhibitors of membrane water permeability, such as mercury, gold, silver, copper, phloretin, tetraethyl ammonium salts, and acetazolamide compounds [3]. Thus, studies of mite aquaporins allow both precise biophysical measurements of their functions and exploration of high-affinity inhibitors for control of these insects.

AQPs are highly conserved proteins across diverse species. The completed Human Genome Project acknowledges 13 AQP members (AQP0–12), which are divided into three subgroups based on their structure, function, cellular location, and permeability: (1) orthodox AQPs (AQPs 0, 1, 2, 4, 5, 6, and 8), which have exclusive water permeability; (2) aquaglyceroporins (AQPs 3, 7, 9, and 10), which are permeable to water, glycerol, and possibly other small polar molecules; and (3) superaquaporins (AQPs 11 and 12) [4]. More classes of AQPs can be found in older lineages of mammals (Metatheria and Prototheria), including AQPs 13 and 14 [5]. The construction of an insect phylogenetic tree by using clustalX2.1, MEGA7.0, Jalview, and Mesquite software classified 45 insect AQPs into four major paralogs, with two conserved Asp–Pro–Ala (NPA) motifs located in the central and C-terminal domains [6].

The AQP primary sequences contain six membrane-spanning segments (TM1–6) with five connecting loops (A–E). Each AQP monomer consists of an extracellular and cytoplasmic vestibule connected by a central amphipathic pore containing hydrophilic and hydrophobic surfaces. The hydrophilic surface comprises alpha-carbonyl groups from the polypeptide backbone, including NPA motifs, and the hydrophobic residues of the pore prevent the permeation of larger molecules. The extracellular vestibule contains an ar/R constriction region or selectivity filter (SF), which comprises aromatic and arginine residues and is also the narrowest region of the pore [7].

Three-dimensional (3D) structural research provides further insights into the atomic-level mechanisms of water and solute conduction and of proton and/or ion exclusion. However, the AQP structure is classically difficult to characterize because purification and crystallization are impeded in a membrane environment. Further, no current experimental methods can monitor permeation through AQPs on a molecular level with sufficient spatial and temporal resolution. Molecular dynamics simulations (MDS) can go beyond static structures, thus offering an ideal method to understand the working mechanism of water and glycerol transport through AQPs at atomic resolution. On the basis of our reports on human AQP4 [8], PfAQP from the malarial parasite *Plasmodium falciparum* [9], and Aqy1 from *Pichia pastoris* [10], the setup for these simulations typically involves a box containing the protein embedded in a simple membrane surrounded with water.

Elucidating the structure of AQPs provides a unique opportunity to understand the mechanisms of these biomolecular systems. By using RNA sequencing and RT-PCR, we identified six AQPs from the common house dust mite *Dermatophagoides farinae*, namely, DerfAQP1–4, DerfAQP5.01, and DerfAQP5.02 (GenBank Nos. KY231248, KY231249, KY231250, KY231251, KY231252, and KY231253) [11]. To better understand its structure and role in mite physiology and pathology, we constructed the tertiary structure and conducted MDS for DerfAQP1.

## 2. Materials and Methods

### 2.1. System Preparation

Templates of the DerfAQP1 (GenBank Accession No. ATL75744.1) protein were analyzed for homology modeling in the Protein Data Bank (PDB) [12] by using Protein BLAST. The appropriate templates were selected based on high score, low *e* value, and maximum sequence identity. The tertiary structure of DerfAQP1 was constructed with Modeller 9.0 (https://salilab.org/modeller). PROCHECK [13] was employed to determine the stereochemical quality of the DerfAQP1 protein structure by analyzing the residue-by-residue geometry and overall structural geometry. VERIFY 3D [14] was used to detect the compatibility of an atomic model (3D) of DerfAQP1 protein with its own amino acid sequence (1D). ERRAT [15] was used to analyze the statistics of nonbonded interactions between different atomic types. QMEAN [16] was used to derive both global (the entire structure) and local (per residue) absolute quality estimates based on a single model. The GMQE [17] score was expressed as a number between 0 and 1, which reflects the expected accuracy of a model built with that alignment and template, and the coverage of the target wherein higher numbers indicate higher reliability. The interactive visualization and analysis of molecular structures was completed using UCSF Chimera 1.13.1 [18].

We used the automatic protein structure file (PSF) builder plugin of the VMD software to generate the PSF and PDB files for the monomeric DerfAQP1 and crystallographic water molecules and ions. The protonation states of all residues were selected to conform to the physiological pH range of 7.4 according to the pKa values of their side chains using PSF builder. To solvate the protein, we used the SOLVATE program [19] to fill any empty space inside the pores and to surround the protein with water. By using the VMD software and our previous scripts [20], the all-atom model system for the DerfAQP1 monomer was formed approximately along the *z*-axis [21] at the center of an 80 × 80 × 100 Å^3^ water box. We then added 52 Na^+^ and 55 Cl^−^ ions to neutralize and salinize the system to 150 mM, with a total of 59,914 atoms.

### 2.2. DerfAQP1 Simulations

Simulations were performed using the Chemistry at Harvard Macromolecular Mechanics 36 (CHARMM36) force field [21, 22], TIP3P water model [23], and molecular dynamics program NAMD version 2.9 software [24] with periodic boundary conditions at a constant temperature of 298 K and constant pressure of 1 atm (NPT ensemble) to conform to standard laboratory conditions. The vDW interactions were calculated with a cutoff distance of 1.0 nm, switching distance of 0.9 nm, and pair-list distance of 1.2 nm. The Langevin dynamics and piston methods [25] were used to maintain constant temperature and pressure. Full electrostatics were employed with the particle mesh Ewald method [26]. Time steps of 2.0 and 1.0 fs were used for long-range electrostatic and for short-range nonbonded and bonded interactions, respectively. The energy of the bulk water molecules of the system was minimized, while protein and ions were fixed. Next, we fixed alpha-carbons on the central parts of the helices (−10 Å < *z* < 10 Å) during the equilibrium molecular dynamics run to preserve the backbone structure [20].

### 2.3. Computation of Water Density in Each Interval through DerfAQP1

To compute the density of water *ρ*(*z*_*i*_) at every interval [*z*_*i*_, *z*_*i*_ + Δ*z*] along the channel, we first obtained a time average of the amount of water molecules within that interval using the following equation:

(1) $$\begin{array}{c} N\left(z_{i}\right)=\frac{\sum_{j=0}^{t_{f}/\Delta t}N\left(z_{i},t_{j}\right)}{t_{f}/\Delta t}, \end{array}$$

where *t*_*f*_ = 20 ns is the duration of the simulation and Δ*t* = 1 ps is the time interval between each frame of the trajectory. We then divided by the length of the interval using the following equation:

(2) $$\begin{array}{c} \rho \left(z_{i}\right)=\frac{N\left(z_{i}\right)}{\Delta z}. \end{array}$$

Thus, we obtained the number of water molecules per unit Angstrom.

### 2.4. Free Energy Computation

As stated by Wambo et al. [27], considering that the simulation is performed in the isobaric−isothermal ensemble, the density function of water along the channel can be considered proportional to the probability distribution function of the ensemble:

(3) $$\begin{array}{c} \rho \left(z_{i}\right)\propto e^{\frac{-\Delta G\left(z_{i}\right)}{k_{B}T}}, \end{array}$$

where Δ*G*(*z*_*i*_) is the Gibbs free energy at point *z*_*i*_. If Δ*G*(*z*_*A*_) ≡ 0 at a reference point *z*_*A*_, the free energy profile along the channel may then be expressed as follows:

(4) $$\begin{array}{c} ∆G\left(z_{i}\right)=k_{B}T\;\ln\left[\frac{\rho \left(z_{A}\right)}{\rho \left(z_{i}\right)}\right]. \end{array}$$

## 3. Results

### 3.1. Preparation and Equilibrium of the MDS System

**Figure 1:**
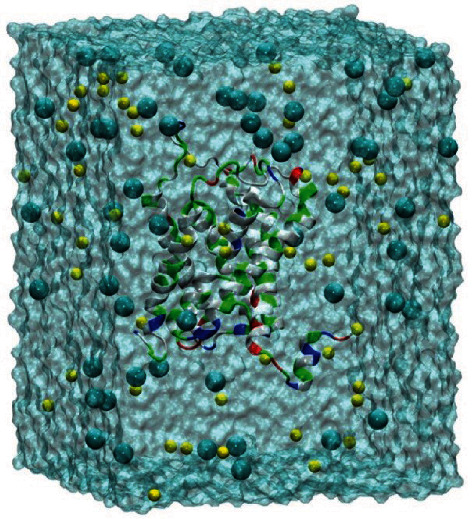
Snapshot of the last frame of the molecular modeling system for DerfAQP1. Builder program provided within the VMD software. Chloride is shown in the indigo blue ball model, sodium is shown in yellow, and proteins are represented by cartoons. Residue type is represented by color: hydrophobic in white, polar hydrophilic in green, negatively charged in red, and positively charged in blue. The water box drawn in MSMS provides VMD fast surface calculation and display with a size of 80 × 80 × 100 Å^3^ and a total of 59,914 atoms.

After searching with Protein BLAST, 6QZI, 6F7H, and 3C02 were selected as templates for the DerfAQP1 homology modeling with sequence identities of 35.59%, 35.63%, and 34.89%, respectively, which meet the requirements of homology modeling. The DerfAQP1 protein's SAVES evaluation results show that its PROCHECK, ERRAT value, and VERIFY 3D results are all ideal, indicating that the results of the DerfAQP1 homology modeling are acceptable (Table 1). The GMQE value of the DerfAQP1 protein is between 0 and 1 and is close to 1, indicating a high reliability of the three protein structures (Table.  1). The QMEAN *Z*-scores showed a good agreement between the DerfAQP1 protein structure and the template protein structure (Table 1). Accordingly, we determined the reliability of the tertiary structure of DerfAQP1 obtained by homologous modeling. By using the automatic PSF builder and Add Solvation Box plugins in the VMD software, the monomer DerfAQP1 was placed in an 80 × 80 × 100 Å^3^ box, and the TIP3P water model and 52 Na^+^ and 55 Cl^−^ ions were added throughout the system. The system consisted of a total of 59,914 atoms (Figure 1).

**Figure 2:**
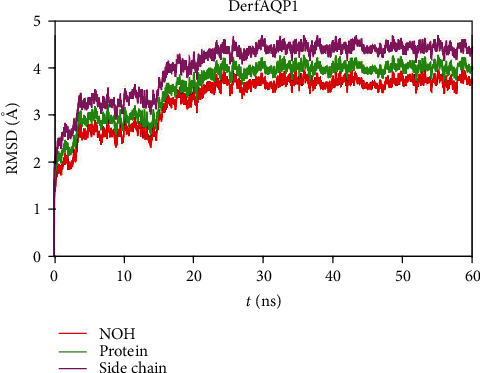
The root mean square deviation of the monomer DerfAQP1 during molecular dynamics simulations. The change with time of the protein without hydrogen atoms, the whole protein, and the protein side chains is shown in red, green, and purple, respectively.

According to the changes of the root mean square deviation (RMSD) of the whole protein, protein without hydrogen atoms, and protein side chains (Figure 2), DerfAQP1 monomers exhibited significant fluctuation at the beginning of simulations but reached a conformational steady state after 30 ns with a RMSD of 3.8–4.2 Å. We saved the files for free energy computation.

### 3.2. Conduction Channel, SF, and NPA Motifs

**Figure 3:**
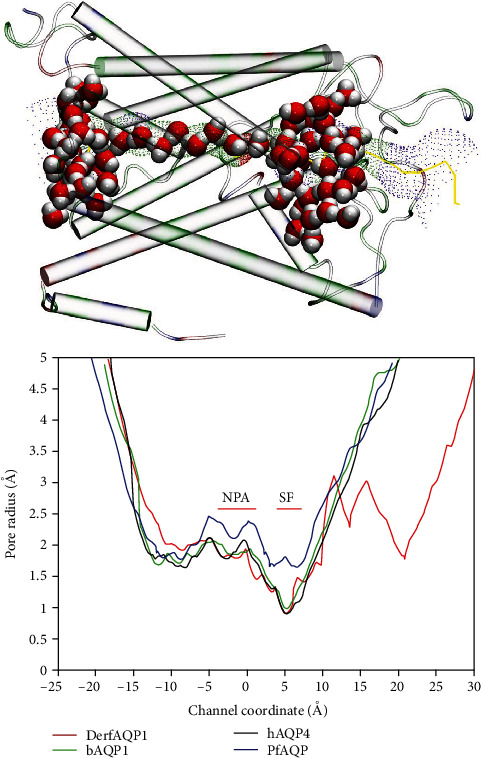
Radius of the DerfAQP1 channels. The top image shows the 3D structure in the cartoon model. The colors represent residue types, and the water molecules are shown in the ball models. Hydrogen atoms are shown in white, while oxygen atoms are shown in red. The channel is shown in green. The selectivity filter (SF) structure, which is the narrowest area in the channel, is shown in red. The bottom image shows the radii comparison among DerfAQP1, bAQP1, hAQP4, and PfAQP, where the SF and NPA regions are marked. Image was drawn in VMD [28] and Hole 2.0 [29].

Comparison of the radii of DerfAQP1 at the equilibrated state to the crystal structures of bAQP1 (PDB code No. 1J4N), hAQP4 (PDB code No. 3GD8), and PfAQP (PDB code No. 3C02) revealed differences between the DerfAQP1 and PfAQP radii, although the radius of DerAQP1 was similar to that of bAQP1. Through the channel, the narrowest site was located on the SF. The two most conserved NPA motifs formed a canonical “fireman's grip-like” structure (Figure 3).

### 3.3. Water Arrangement in the Channel

**Figure 4:**
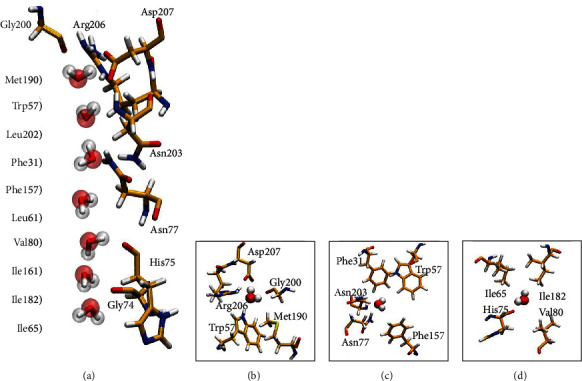
The water-conducting channel of one monomer of DerfAQP1: (a) the hydrophilic residues are shown in licorice, hydrogen in white, oxygen in red, carbon in yellow, and nitrogen in blue. The licorice ball models represent water, (b) residues around the selectivity filter structure; (c) residues around the NPA structure; (d) residues between the cytoplasm and channels.

In the last frame, seven water molecules formed a single file in the channel of the DerfAQP1 monomer (Figure 4(a)). Before the water molecules entered the channel, they stayed in the extracellular vestibule, which contained Arg206, Trp57, Met190, Gly200, and Asp207 (Figure 4(b)). The middle section of the DerfAQP1 channel contained two conserved NPA motifs similar to that in bAQP1 and hAQP4 (Figure 4(c)), resulting in a narrower radius than the channel of PfAQP containing Asn–Leu–Ala and Asn–Pro–Ser [30]. In the channel near the cytoplasm, the water molecules interacted with residues His75, Val80, Ile65, and Ile182 (Figure 4(d)).

### 3.4. Free Energy Computation

**Figure 5:**
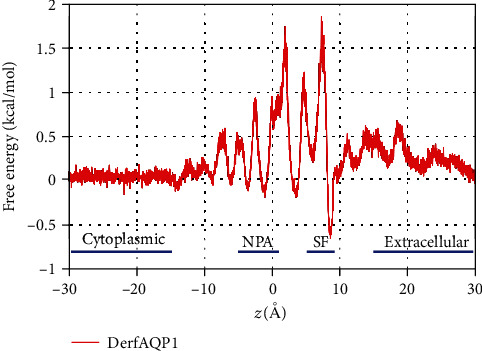
The free energy profile of water permeation through the channels of DerfAQP1. The cytoplasmic side, Asn–Pro–Ala, selectivity filter, and extracellular side are marked on the free energy curve at the *z*-axis of the channel. The free energy on the starting position was recorded as zero.

Considering that the single file in the channel of the DerfAQP1 monomer is located between –10 Å and 10 Å (–10 Å < *z* < 10 Å), the free energy profile showed an energy barrier of ~2.5 kcal/mol with respect to the SF structure (Figure 5) [31]. As we described previously [20], the error associated to our free energy calculation originates from the thermal fluctuation energy kBT = 0.6 kcal/mol inherent to molecular dynamics simulations. Since we are subtracting two measurements of energy at different points of the channel, this becomes ±1.2 kcal/mol.

## 4. Discussion

The current study investigated the working mechanisms of house dust mite AQP to provide information about the future development of AQP inhibitors. MDS showed that seven water molecules were arranged in a single file through the pores of DerfAQP1. As the water molecules trickled through the pores, their polarization changed around the NPA motifs in Asn203 and Asn77. Residues Arg206, Trp57, Met190, Gly200, and Asp207 constitute the extracellular vestibule of the pore, while residues His75, Val80, Ile65, and Ile182 constitute the cytoplasmic portions. Therefore, these key amino acid residues should be the targets for the development of new inhibitors, which can be designed and tested in silico against the docking energies of AQP4 [32].

In 2004, Law and Sansom performed MDS (totaling almost 40 ns) by using the X-ray structures of Aqp1, a homology model of Aqp1 based on the GlpF structure, and the intermediate resolution structures of Aqp1 derived from the electron microscopy [33]. These results suggest that the homology models based on bacterial homologues could be used to derive functionally relevant information on the structural dynamics of mammalian transport proteins. Here, we performed MDS of DerfAQP1 with homology modeling constructed from the X-ray structure of malaria PfAQP (PDB code No. 3C02) [30]. Subsequently, we placed the monomer DerfAQP1 in an 80 × 80 × 100 Å^3^ box and added the TIP3P water model and 52 Na^+^ and 55 Cl^−^ ions throughout the system. The RMSD change in the last few nanoseconds of the MDS is insufficient to demonstrate our success.

All identified AQP structures have a common homotetrameric arrangement, and each AQP monomer folds into a right-handed alpha-barrel architecture. Our monomer AQP model in simulation achieved structural equilibrium after 30 ns according to the RMSD. Moreover, the free energy profile showed an energy barrier of ~2.5 kcal/mol, and this condition was observed in the SF structure, which was the narrowest part of the pore. Computational free energy profiles deliver valuable structure–function relationships in regard to selectivity. For example, the free energy profiles for the computational mutants of two channels, Aqp1 and GlpF, where the bipolar field was eliminated by artificially discharging backbone atoms [34], suggested that mutating the energy barrier (i.e., SF structure) may effectively destroy mite AQPs.

## 5. Conclusions

We conducted MDS of the mite AQP DerfAQP1 and computed its free-energy profile for the first time. We are conducting similar simulations for other mite AQPs. The analysis of water molecules through the water channel can further elucidate the AQP structure and functional implications. The structural comparison of mite AQPs, such as mutating the key amino acid residues, can also be helpful in the development of inhibitors for mite control.

## Figures and Tables

**Table 1:** Evaluation of the tertiary structure of DerfAQP1.

Protein	Structural assessment method	PROCHECK (%)	ERRAT value	VERIFY 3D (%)	GMQE score	QMEAN *Z*-score
DerfAQP1	SAVES	88.9 core	93.25	86.93		
		9.6 allowed, 1.2 generously allowed, 0.2 disallowed				
	GMQE				0.73	
	QMEAN					−3.9

## Data Availability

The data used to support the findings of this study are available from the corresponding author upon request.
